# New Appliances and Things Medical

**Published:** 1894-11-03

**Authors:** 


### NEW APPLIANCES AND THINGS MEDICAL.

### ROYAL SELTERS NATURAL TABLE WATER.

(Siemens and Co. Agents: Pickhardt and Co., 26, Regent Street.)

These fine mineral waters are perhaps hardly as well known in England as their valuable therapeutic qualities warrant. The composition of the water closely resembles that of the famous Ems spring, and hence Selter Water is not only pleasant to the palate, but it is also invaluable for those suffering from derangement of the stomach or bowels consequent on a gouty diathesis. By a concession from the German Government the firm of Siemens and Co. have the monopoly of this water, and without any artificial treatment the water is bottled direct from the spring. The water contains a very high percentage of free and combined carbonic acid, and hence is sparkling and effervescent when freshly uncorked. Being only slightly aperient and diuretic it may safely be used by those in good health, and constitutes a fine sparkling water for domestic use, and whether taken by itself or after the addition of wine, spirits, or fruit syrups, it is always certain to recommend itself to its patrons.

### "IDEAL" BREAD.

(Peter Mumford, Royal Flour Mills, Vauxhall, S.E.)

This new departure in the manufacture of bread is due to the development of an original process discovered and provisionally protected under the Patent Acts by Mr. J. T. O'Callaghan. The bread itself is prepared from meals which are known as the Peptic "Ideal" Meals. These meals are prepared by an elaborate and ingenious treatment of wheat, and constitute most valuable material from which to manufacture a bread which, besides being exceedingly palatable, is nourishing and digestible, and peculiarly adapted for invalids, children and persons of enfeebled digestive powers, containing as it does the valuable properties of the germ and wholemeal. It further has the advantages of retaining its moisture for a considerable period, and of very ha pily lending itself to the cutting of thin bread and butter. The meal is rich in lime and potash salts, and consequently is valuable in the synthesis of bone and muscle during the growing years of childhood. We have every confidence in recommending "Ideal" bread as a pure and wholesome substitute for the ordinary household variety.

